# Specific absorption rate (SAR) simulations for low-field (< 0.1 T) MRI systems

**DOI:** 10.1007/s10334-023-01073-3

**Published:** 2023-03-18

**Authors:** Javad Parsa, Andrew Webb

**Affiliations:** 1 C.J. Gorter MRI Centre, Department of Radiology, Leiden University Medical Center, Leiden, The Netherlands; 2 Percuros B.V., Leiden, The Netherlands

**Keywords:** Low field MRI, Specific absorption rate, Transmit efficiency, Electromagnetic simulations, Point-of-care MRI

## Abstract

**Objective:**

To simulate the magnetic and electric fields produced by RF coil geometries commonly used at low field. Based on these simulations, the specific absorption rate (SAR) efficiency can be derived to ensure safe operation even when using short RF pulses and high duty cycles.

**Methods:**

Electromagnetic simulations were performed at four different field strengths between 0.05 and 0.1 T, corresponding to the lower and upper limits of current point-of-care (POC) neuroimaging systems. Transmit magnetic and electric fields, as well as transmit efficiency and SAR efficiency were simulated. The effects of a close-fitting shield on the EM fields were also assessed. SAR calculations were performed as a function of RF pulse length in turbo-spin echo (TSE) sequences.

**Results:**

Simulations of RF coil characteristics and B_1_^+^ transmit efficiencies agreed well with corresponding experimentally determined parameters. Overall, the SAR efficiency was, as expected, higher at the lower frequencies studied, and many orders of magnitude greater than at conventional clinical field strengths. The tight-fitting transmit coil results in the highest SAR in the nose and skull, which are not thermally sensitive tissues. The calculated SAR efficiencies showed that only when 180° refocusing pulses of duration ~ 10 ms are used for TSE sequences does SAR need to be carefully considered.

**Conclusion:**

This work presents a comprehensive overview of the transmit and SAR efficiencies for RF coils used for POC MRI neuroimaging. While SAR is not a problem for conventional sequences, the values derived here should be useful for RF intensive sequences such as *T*_1ρ_, and also demonstrate that if very short RF pulses are required then SAR calculations should be performed.

**Supplementary Information:**

The online version contains supplementary material available at 10.1007/s10334-023-01073-3.

## Introduction

The development of low-field point-of-care (POC) MRI systems has grown significantly in recent years [1, 2], with both academic and commercial groups active in this endeavor [3–21]. Many in vivo studies using POC systems have been performed on C- or H-shaped permanent magnet arrangements, either using a commercial Hyperfine unit [3–7, 12] or custom-built research systems [20, 21]. The other major geometry used for in vivo POC studies is Halbach-based, either with an inbuilt gradient [8, 9, 11] or a homogeneous field [14, 17–19, 22]. Field strengths vary from ~ 0.05 to 0.1 T for these types of POC neuroimaging systems.

Under conditions in which tissue conductivity is frequency independent, and the RF skin depth is large compared to the dimensions of the object, then the specific absorption rate (SAR) scales as the square of the frequency [23], and so the SAR at 50–100 mT is many hundreds of times lower than at 1.5 T, for example. Indeed, most publications simply state that SAR is much less or even not a problem at low field. However, in practice, there are limiting issues, based on the geometry of POC systems, which may reduce this factor of many hundreds. Due to limited space, the RF transmit coils are typically tight fitting around the head, which means that the strong local electric fields very close to the coil conductors can penetrate into the skull. Most RF coils are variations on a solenoidal geometry, which produces relatively high conservative electric fields. While this effect can be reduced by increasing the diameter of the RF coil, the limited space within POC systems means that the gap between the RF coil and RF shield would then become extremely small, significantly decreasing the transmit efficiency of the coil and requiring more power to be supplied, as well as decreasing the signal-to-noise ratio (SNR). An additional factor is that the B_0_ homogeneity over the brain in POC systems is many orders of magnitude lower than for conventional whole body superconducting clinical magnets, and, therefore, shorter RF pulses are typically required to excite the relatively large bandwidth of the proton resonances: shorter pulses require higher B_1_^+^ strength for a given tip angle, and this results in an increased SAR. Even considering all of these factors, SAR is still much less than at clinical field strengths, but given that one of the advantages of low field is that high B_1_^+^ fields can be used, for example, to produce long echo trains in turbo-spin echo (TSE) sequences with full 180° refocusing pulses, as well as high power pulses for magnetization transfer (MT) and spin-lock (*T*_1ρ_) contrast, it is important to have quantitative measures of SAR even at low field.

A few studies on SAR at low fields have been reported. Hayden et al. [24] performed an experimental study to estimate the average SAR over the body over a frequency range of 30 kHz to 1.25 MHz. They used a technique in which body losses were estimated via changes in the quality (*Q*) factor of the RF coil when the volunteer was positioned inside [25, 26]. They specifically considered the case of a linearly polarized time-varying B_1_^+^ field applied normal to the sagittal plane of the human body. Their results showed that the average SAR, as predicted, scaled as the square of the frequency over this frequency range. As they noted, their measurements estimate average SAR rather than localized measurements. Van Speybroek et al. [18] performed numerical simulations based on the analytical equations derived by Bottomley et al. for perfectly uniform excitation fields [27], and showed how the SAR varies with inter-pulse time and pulse length in TSE sequences.

In this paper, we performed electromagnetic (EM) simulations at four different fields 47 mT (2.0 MHz), 64 mT (2.7 MHz) relevant to Hyperfine systems, 75 mT (3.2 MHz) and 100 mT (4.3 MHz), using two different RF coil geometries, a semi-elliptical helix [28] and a circular saddle geometry, both designed for adult neuroimaging. Both coils were designed to be very close fitting to the head, as is the practical case for the limited space available on POC systems. The parameters characterized were the transmit magnetic field efficiency (B_1_^+^ per square root Watt input power), transmit electric field for the same input power, SAR (both 1 g averaged and 10 g averaged), and SAR efficiency (B_1_^+^ divided by the square root of the maximum SAR). Experimental B_1_^+^ maps were acquired to compare with simulation results. In addition, the effects of an RF shield were quantified in terms of transmit efficiency and SAR efficiency. Finally, we also computed 10 g and whole head SAR for TSE sequence with different scan parameters.

## Materials and methods

### Electromagnetic simulations

**Fig. 1:**
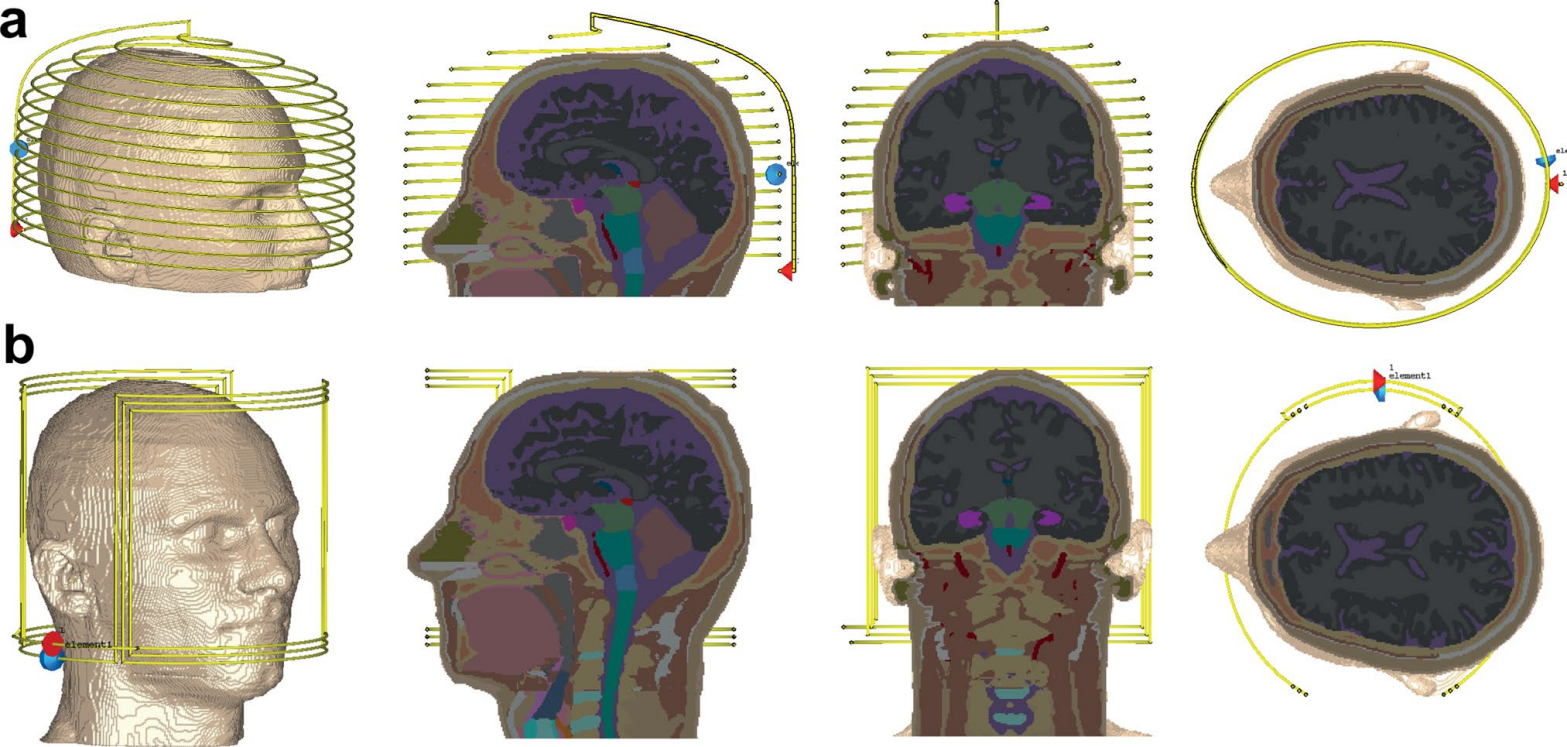
Schematics of the simulated semi-elliptical helix coil, **a** and saddle coil, **b** with sagittal, coronal, and transverse cross-sections of the human head model

Electromagnetic (EM) simulations were performed in CST Microwave Studio (CST GmbH, Darmstadt, Germany). Two RF coils were simulated. The first was a semi-elliptical helix, consisting of 15 turns of copper wire of 1.5 mm diameter, with one capacitive segmentation halfway along the wire length. The coil was tuned to its respective Larmor frequency using a lumped capacitor (*C*_t_) positioned at the mid-turn and matched to 50 Ω using a single capacitor (*C*_m_) at the input port in series with an ideal voltage source. The coil had dimensions 180 mm width, 240 mm height, and 170 mm depth. For simulations to determine the effect of the RF shield, the shield was 300 mm in diameter with a length of 350 mm and a copper thickness of 60 mm. The second coil was a saddle geometry with 200 mm diameter and 180 mm length, consisting of three turns of 1.5 mm diameter copper wire and one capacitive segmentation at the centre. A virtual 34-year-old male model, Duke, from the IT IS Virtual Family was positioned inside the RF coil and only the head was included in this simulation [29]. The center of the pineal gland was taken as the center of the brain. With respect to this point, the centre of the coil is 15 mm toward the bottom of the head and 25 mm toward the front of the head. These displacements correspond to the physical situation in our Halbach array system. Figure 1 shows the setups for the EM simulations for the two different RF coil geometries with model inside. The head model has an isotropic resolution of 1 mm × 1 mm × 1 mm and contains 196 × 310 × 176 voxel elements. This model is categorized into more than 40 different tissue types with 25 dielectric property values. To simplify the number of dielectric properties in the model, the same electric conductivity and permittivity were considered for tissues in the same category (see supplementary Table 1), which led to 18 different dielectric values for the tissues; for example the eye, sclera, cornea, and vitreous were considered to have the same EM properties. To accelerate the simulation by reducing number of mesh cells, only the head part of model was considered in simulation (if the torso is also included then results showed a very small difference of ~ 4%). Electric and open boundary conditions were set in all directions, and the computations were ended at an accuracy of − 40 dB. The time domain solver was used with 1,960,000 hexahedral mesh elements and 1 W input power was considered for all simulations.

Although conductivity varies very little over the frequency range considered here [24], the large variation in efficiency of dielectric relaxation mechanisms at low frequencies means that the relative permittivity does change significantly, with higher values at lower frequencies [30]. Table 1 lists the relevant parameters for the main brain constituents (white matter, gray matter and cerebrospinal fluid) used in the simulations.

**Table 1:** Parameters used in the electromagnetic simulations [30]

Magnetic field strength (mT)	47	63.5	75	100
Larmor frequency of ^1^H (MHz)	2	2.72	3.19	4.25
Permittivity				
White matter	341	297	278	248
Gray matter	656	586	552	492
CSF	109	109	109	109
Conductivity (S/m)				
White matter	0.112	0.117	0.12	0.127
Gray matter	0.18	0.19	0.2	0.27
CSF	2	2	2	2

For each field strength, the coil reflection coefficient (*S*_11_), B_1_^+^field, E field, SAR (SAR_1g_) averaged over 1 g of tissue, SAR (SAR_10g_) averaged over 10 g of tissue, transmit efficiency and SAR efficiency were calculated.

### *Experimental B*_*1*_^+^*mapping*

In order to validate the simulation data, a B_1_^+^ map from the semi-elliptical helix coil was acquired at 47 mT and compared with simulation data at the same frequency. The magnet, gradient and RF coil system have been extensively described in the literature [14, 22]. A three-dimensional double angle mapping (DAM) method was used [31] with two 3D gradient echo (GRE) sequences with flip angles of 60° and 120°, TR/TE 500 ms/6 ms, field-of-view 200 × 200 × 200 mm^3^, ≈ 3 × 3 × 3 mm^3^ spatial resolution, acquisition bandwidth 50 kHz over FOV, and RF pulse duration 100 µs. The B_1_^+^ maps were calculated on a pixel-by-pixel basis from the corresponding tip angle (*α*) maps using the formula:

1 $$\alpha ={\mathrm{cos}}^{-1}\left(\frac{{S}_{2}}{2{S}_{1}}\right)=\gamma {B}_{1}^{+}\tau ,$$

where *S*_1_ and *S*_2_ are the signal intensity of images acquired 60° and 120°, respectively, and $\tau$ is the RF pulse duration time. The data were acquired from a head phantom filled with copper sulphate doped water with *T*_1_ ≈ 140 ms.

## Results

### Comparison of simulated and experimental RF coil characteristics

**Fig. 2:**
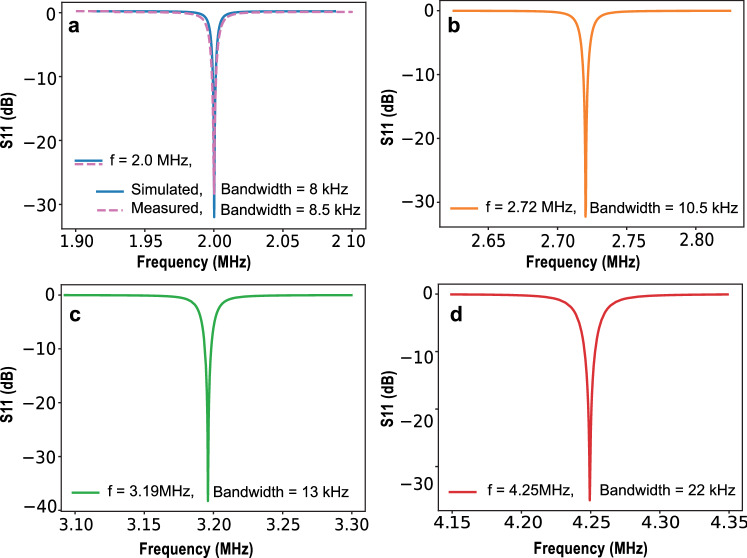
Simulated *S*_11_ at four field strengths (frequencies) showing reflection coefficients less than − 30 dB. **a** Comparison between simulated *S*_11_ and measured *S*_11_

Simulated S_11_ reflection coefficients of the semi-elliptical spiral RF coils at the four different frequencies are shown in Fig. 2, which shows almost 99% of the power transmitted to the coil. Simulations were compared with experimental measurements for the coil at 47 mT, showing good agreement.

### Simulations of magnetic and electric fields

**Fig. 3:**
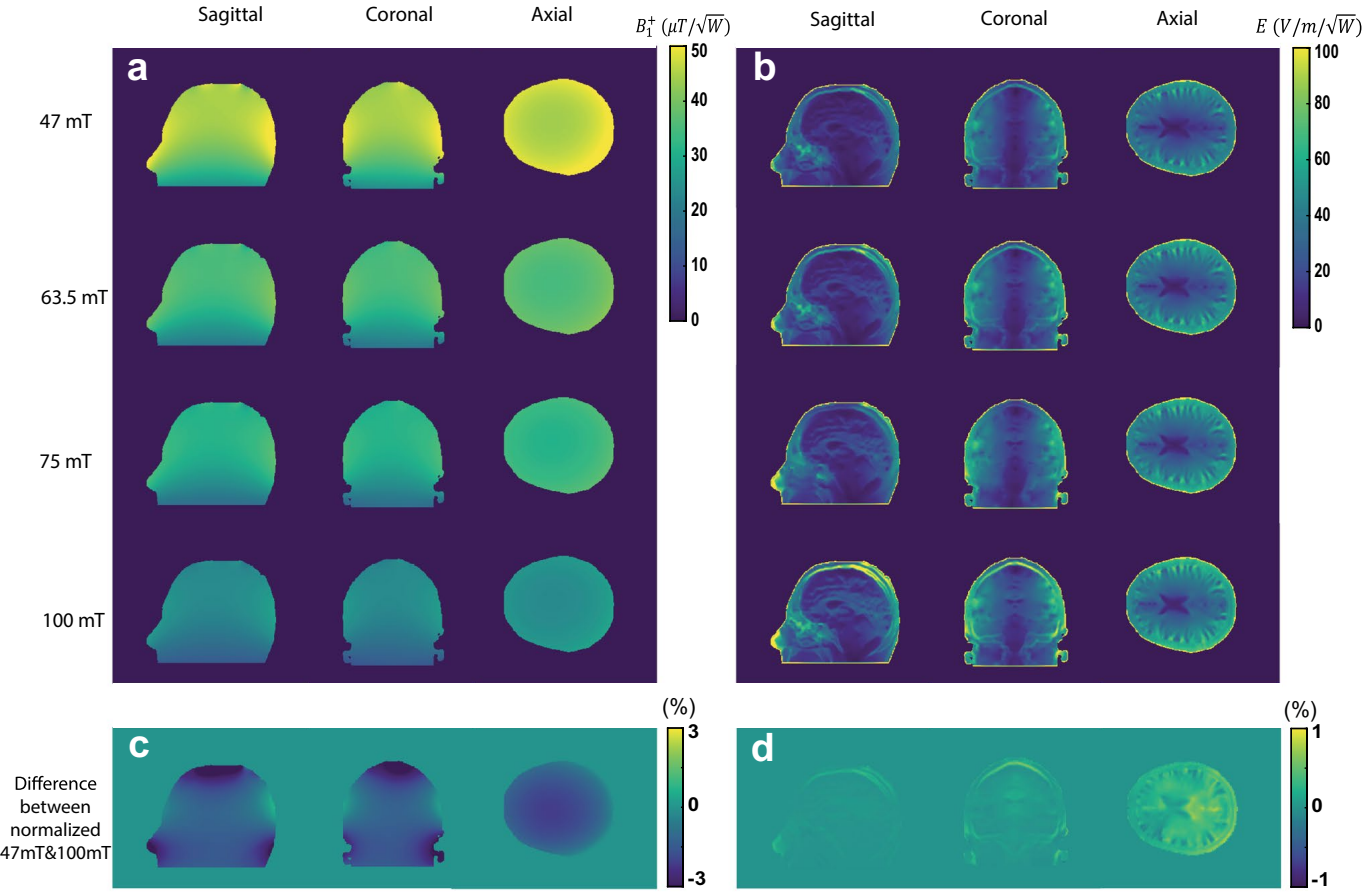
**a** Simulated transmit efficiency (B_1_^+^ per square root input power) in three central planes in the head model. The axial plane was positioned at the centre of the brain slightly above the eyes. **b** Corresponding electric field distributions calculated per square root input power. **c**, **d** difference between the normalized map of 47 mT and 100 mT in percent (each map is normalized with respect to its maximum value)

Figure 3 shows the simulated B_1_^+^ efficiency (microTesla per square root Watt input power) and electric field (Volts/metre per square root Watt input power) for the semi-elliptical spiral coil for the four different frequencies. The B_1_^+^ efficiency patterns show the expected high homogeneity throughout the brain region, with higher values very close to the conductor surfaces and a gradual drop-off in the head-foot direction at the end of the coil. The transmit efficiency drops as a function of frequency, but the relative distribution is almost same over all frequencies. Normalized difference maps shows only a 3% spatial variation between the maps obtained at 47 mT and 100 mT. In terms of the electric field, the pattern is also similar across all frequencies (only 1% difference in the spatial distribution), with the absolute values increasing with frequency.

### Comparison of simulated and experimental transmit magnetic fields

**Fig. 4:**
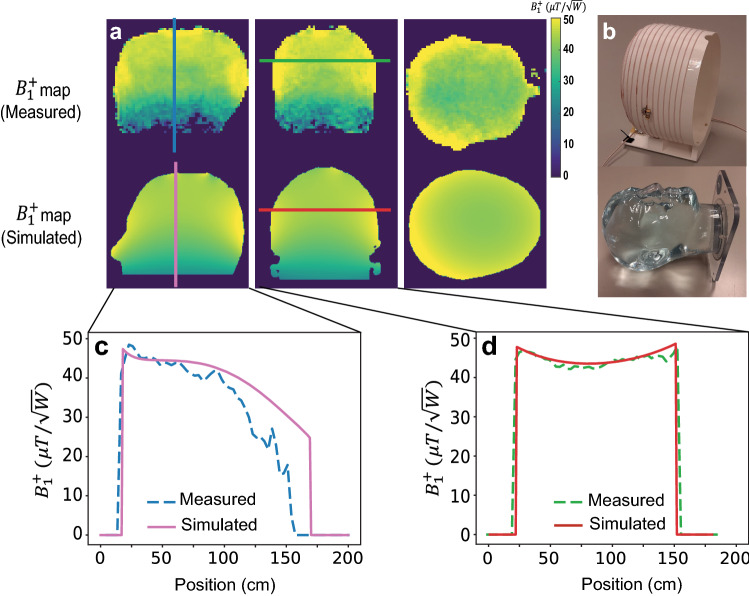
**a** A comparison between simulated and measured transmit efficiency in three central planes. **b** Phantom and constructed coil. **c**, **d** The corresponding 1D plots in the sagittal and coronal planes

Figure 4 shows the measured and simulated B_1_^+^ maps in three orthogonal planes at 47 mT. The one-dimensional projection plots show reasonable agreement between the experimental and simulation data, with a slightly sharper drop-off in the head–foot direction seen in the experimental data.

### Simulations of SAR and SAR efficiency

**Fig. 5:**
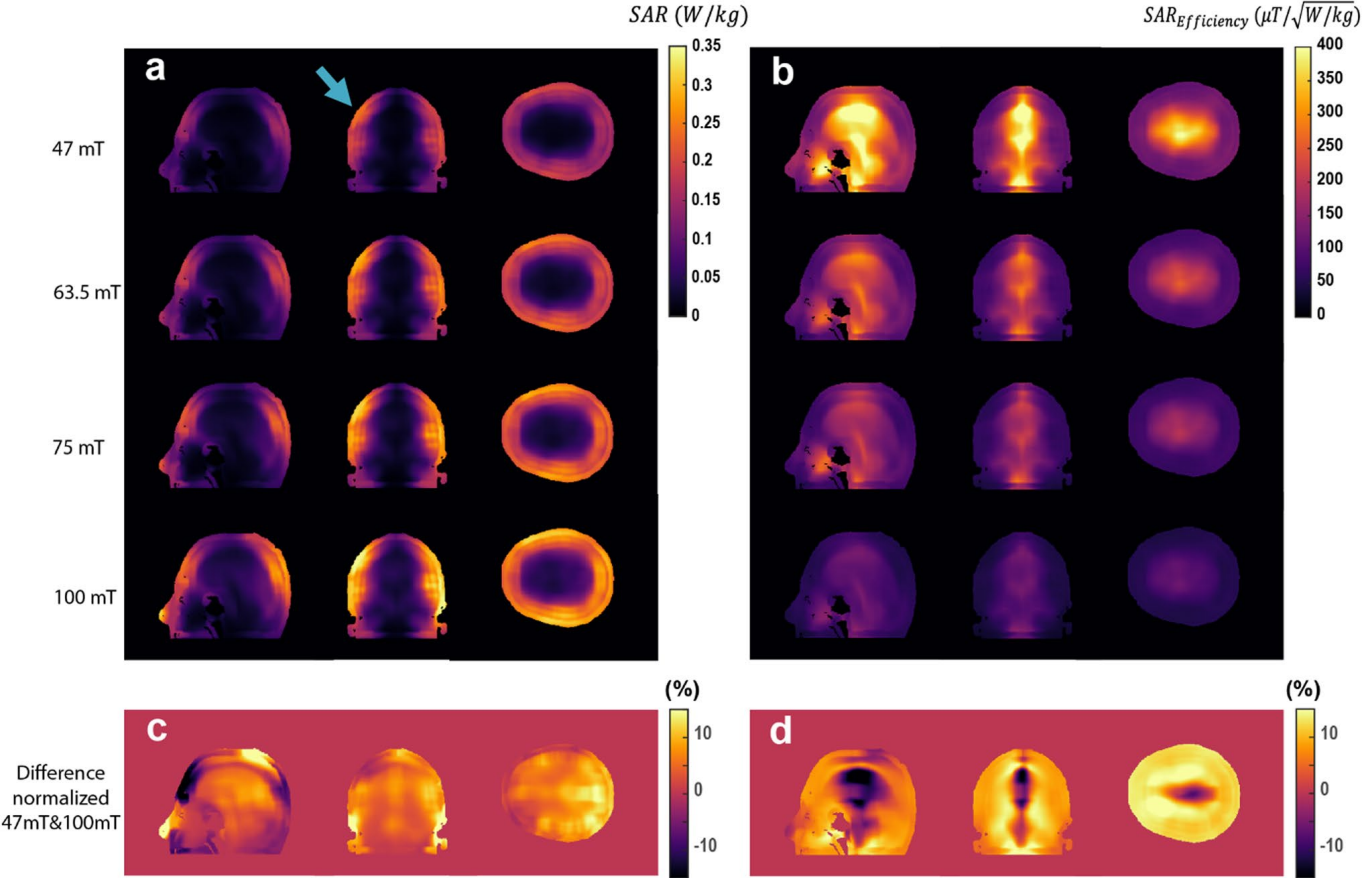
**a** 10 g-averaged SAR map per 1 W of coil input power through the slices in orthogonal planes which contain the highest SAR values. **b** SAR efficiencies corresponding to each 10 g-averaged SAR map on the left. **c**, **d** Difference between the normalized map of 47mT and 100 mT in percent (each map normalized to its maximum)

Figure 5 shows simulated SAR_10g_ and SAR_10g_ efficiencies at the four different field strengths (corresponding figures for SAR_1g_ can be found in the supplementary material). There are regions of elevated SAR very close to the conductors of the RF coil, but even these values are low compared to those found at clinical field strengths. As expected the highest SAR efficiency is in the centre of the brain, where the E field is lowest, and the SAR efficiency decreases with frequency. The values of SAR efficiency, in the several hundreds of microtesla per square root Watts/kg, can be contrasted with typical values reported at 7 T of only 1–2 microtesla per square root Watts/kg [32].

### Simulated SAR for TSE sequences

TSE sequence with a large number of echoes and full 180◦ refocusing pulses can be run at low field because of the lower SAR. Using the equations outlined in Bottomley [27] and used in similar calculations by Van Speybroeck [18], the SAR can be expressed as:

2 $${\mathrm{SAR}}_{\mathrm{TSE}}=\left[\frac{\mathrm{SAR}}{{{(\mathrm{B}}_{1}^{+})}^{2}}\right]\times \frac{{\pi }^{2}}{{\gamma }^{2}\tau \mathrm{TR}}\times \left({\left(\frac{2{\alpha }_{1}}{\pi }\right)}^{2}+\mathrm{ETL}\times {\left(\frac{2{\alpha }_{2}}{\pi }\right)}^{2}\right),$$

where ${\alpha }_{1}$ and ${\alpha }_{2}$ represent the excitation and refocusing tip angles, $\gamma$ the gyromagnetic ratio, *τ* the pulse duration, TR the repetition time, and $\mathrm{ETL}$ the echo train length, which is equal to the number of refocusing pulses. The SAR and ${B}_{1}^{+}$ from previously shown simulations were used as the inputs to equation [2]. In this case, ${\mathrm{SAR}}_{\mathrm{TSE}}$ was computed for all frequencies over the region with the highest SAR_10g_, as denoted by the blue arrow in Fig. 5. The results of this computation are shown in Table 2 for different RF pulse durations. These calculations assumed a TR of 300 ms, which is approximately the *T*_1_ of brain tissue at these frequencies. In addition, the “worst-case” average SAR over whole head using an ETL of 128 was computed as 1.1, 2.2, 3.3, and 6.8 W/kg at 47 mT, 63.5 mT, 75 mT, and 100 mT, respectively.

**Table 2:** SAR estimation for TSE sequence based on 10 g-averaged SAR map with different 180° pulse durations and ETLs

	Pulse length	*τ* = 10 µs			*τ* = 100 µs			*τ* = 1000 µs		
	ETL	8	32	128	8	32	128	8	32	128
SAR (W/kg)										
100 mT		0.62	2.50	10.30	0.06	0.25	1.0	0.006	0.03	0.10
75 mT		0.35	1.41	5.89	0.03	0.14	0.59	0.004	0.01	0.06
63.5 mT		0.25	1.16	4.66	0.02	0.12	0.47	0.003	0.01	0.05
47 mT		0.13	0.52	2.91	0.01	0.05	0.29	0.001	0.005	0.03

In the particular case of neuroimaging on our 47 mT system, we note that the input power required for a 100 ms 90° RF pulse is almost exactly 1 W, and so the values in the table can be used directly.

### Effect of the RF shield

**Fig. 6:**
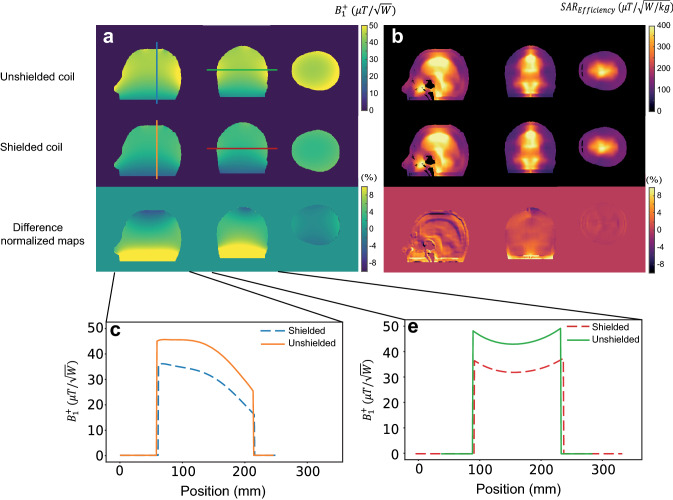
**a** A comparison between transmit efficiency of the coil in unshielded and shielded conditions at three central planes. **b** 10 g-averaged SAR efficiency for both unshielded and shielded coil configurations. **c**, **d** The corresponding 1D-plots in the sagittal and coronal plane

As mentioned previously, the compact nature of head scanners means that the conductors of the RF coil are very close to the inner Faraday shield, and this results in a loss in the transmit efficiency due to the redistribution of the B_1_^+^ field between the shield and RF coil [33]. Simulations were performed using an unshielded coil and one with a circular RF shield with diameter 300 mm, i.e., a 30 mm gap to the RF coil in the long elliptical axis, and a 60 mm distance in the short elliptical axis. Figure 6 shows that the B_1_^+^ efficiency is reduced by approximately 25% by the presence of the shield. However, there are negligible changes in the SAR efficiency.

### Comparisons with saddle coil geometry

**Fig. 7:**
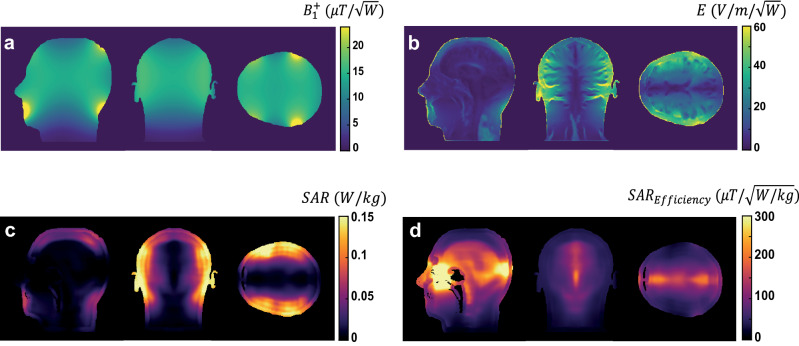
Simulation results for the saddle coil geometry at 4.25 MHz (100 mT scanner). **a** Transmit efficiency, **b** electric field, **c** 10 g-averaged SAR in three slices with the highest SAR and **d** corresponding SAR10g efficiencies. 1 W input power was used for all simulations

Another coil geometry which has occasionally been used for low-field imaging is a saddle or birdcage coil [34]. Although the sensitivity of such coils, which are necessarily operated in linear mode, is lower than that of a solenoid, they are also less sensitive to external noise, and having low inductance also have lower conservative electric fields. Figure 7 shows the simulated transmit efficiency, electric field, SAR_10g_ and SAR_10g_ efficiency for the coil at the highest frequency (4.25 MHz) in this study. The results show a much lower transmit efficiency than the semi-elliptical spiral coil, as expected, but similar SAR efficiency.

## Discussion

This work has simulated a number of different parameters for POC MRI systems with magnet strength ≤ 100 mT fields in a human head model, with values agreeing well with selected experimentally measured parameters. Although not explicitly covered in this paper, the results of the simulations also allow an accurate estimate of the SNR achievable using such coils based on the principle of reciprocity [35].

In this low-frequency regime, the electric field and the SAR are highest toward the outside of the head, with a very low value near the centre of the head. The areas of maximum SAR appeared in regions that were very close to the coil conductors such as the nose and skull, which are not considered to be thermally sensitive structures. These SAR “hot spots” could be minimized by increasing the diameter of the RF coil, but this would also reduce the transmit efficiency due to the presence of mirror currents on the shield.

As expected, the SAR efficiency in this frequency range is orders of magnitude higher than that at clinical field strengths. This confirms the common statements that SAR concerns can be essentially ignored for most POC MRI systems. However, even in this very low-frequency regime, there are limits, for example, in the application to TSE sequences using very short 180° pulses and long echo train lengths. Table 2 results shows in case of using very short RF pulses the SAR does need to be considered carefully.

## Supplementary Information

Below is the link to the electronic supplementary material.

**Supplementary Figure 1**. (a) 1g-averaged SAR map per 1 watt of input power in the three orthogonal planes which show the highest SAR. (b) Corresponding SAR efficiencies. (EPS 8085 KB)

Supplementary file2 (DOCX 23 KB)

## Data Availability

The data that support the finding of this study are available on the request of corresponding author AW.
