# Supplementary material for: Specific absorption rate (SAR) simulations for low-field (< 0.1 T) MRI systems
Source: MAGMA. 2023 Mar 18;36(3):429–38. doi: 10.1007/s10334-023-01073-3 (PMC10386976; doi:10.1007/s10334-023-01073-3)
Supplement: Supplementary file 2 — Supplementary file2 (DOCX 23 KB) [file 10334_2023_1073_MOESM2_ESM.docx]

**Supplementary Table 1** Relative permittivity (ε), electric conductivity (σ), and density of different tissue in the head model used in the electromagnetic simulations. The 40 different physiological tissues in the model were compressed into 18 different tissue groups based on having similar values. In groups where there are differences in ε and σ, an asterix denotes the tissue properties used.

| Tissue Group | Tissue | Permittivity (F/m)  2 MHz 2.72 MHz 3.19 MHz 4.25 MHz | | | | Electric Conductivity (S/m)  2 MHz 2.72 MHz 3.19 MHz 4.25 MHz | | | | Density (kg/m³) |
| --- | --- | --- | --- | --- | --- | --- | --- | --- | --- | --- |
| 1 | Air | 1 | 1 | 1 | 1 | 0 | 0 | 0 | 0 | 1 |
| 2 | Artery* | 168 | 120 | 101 | 720 | 0.92 | 0.97 | 0.99 | 1 | 1050 |
|  | Blood Vessel Wall | 165 | 149 | 142 | 131 | 0.33 | 0.33 | 0.33 | 0.33 | 1102 |
| 4 | Brain  (White Matter) | 341 | 297 | 278 | 248 | 0.11 | 0.11 | 0.12 | 0.12 | 1041 |
| 5 | Cerebellum | 920 | 837 | 794 | 716 | 0.2 | 0.22 | 0.23 | 0.25 | 1045 |
|  | Medulla Oblongata |  |  |  |  |  |  |  |  | 1046 |
|  | Midbrain |  |  |  |  |  |  |  |  | 1046 |
|  | Pons |  |  |  |  |  |  |  |  | 1046 |
| 6 | Ear skin | 848 | 775 | 720 | 630 | 0.03 | 0.4 | 0.05 | 0.08 | 1109 |
|  | Skin* | 858 | 775 | 727 | 634 | 0.03 | 0.05 | 0.06 | 0.09 |  |
| 7 | Cartilage* | 815 | 613 | 525 | 396 | 0.27 | 0.29 | 0.31 | 0.32 | 1100 |
|  | Ear cartilage | 805 | 613 | 525 | 396 | 0.25 | 0.29 | 0.29 | 0.31 |  |
| 8 | Cerebrospinal Fluid | 109 | 109 | 109 | 109 | 2 | 2 | 2 | 2 | 1007 |
| 9 | Connective Tissue | 136 | 129 | 125 | 120 | 0.39 | 0.39 | 0.39 | 0.39 | 1027 |
|  | Tendon |  |  |  |  |  |  |  |  | 1142 |
| 10 | Skull | 106 | 88 | 80 | 65 | 0.02 | 0.03 | 0.03 | 0.03 | 1908 |
|  | Tooth |  |  |  |  |  |  |  |  | 2180 |
|  | Vertebrae |  |  |  |  |  |  |  |  | 1908 |
|  | Mandible |  |  |  |  |  |  |  |  | 1908 |
| 11 | Brain (Grey Matter) | 656 | 586 | 552 | 492 | 0.18 | 0.19 | 0.19 | 0.21 | 1045 |
|  | Hippocampus |  |  |  |  |  |  |  |  |  |
|  | Hypothalamus |  |  |  |  |  |  |  |  |  |
|  | Thalamus |  |  |  |  |  |  |  |  |  |
| 12 | Nerve | 555 | 434 | 382 | 302 | 0.15 | 0.16 | 0.17 | 0.18 | 1075 |
|  | Spinal Cord |  |  |  |  |  |  |  |  |  |
| 13 | Mucous Membrane | 826 | 582 | 489 | 362 | 0.54 | 0.56 | 0.57 | 0.58 | 1102 |
|  | Muscle* |  |  |  |  |  |  |  |  | 1090 |
|  | Tongue | 1150 | 817 | 683 | 495 | 0.45 | 0.48 | 0.49 | 0.52 | 1090 |
| 14 | Commissure Anterior | 341 | 297 | 278 | 248 | 0.11 | 0.11 | 0.12 | 0.12 | 1041 |
|  | Commissure Posterior |  |  |  |  |  |  |  |  |  |
| 15 | Eye (Cornea) | 1430 | 1000 | 836 | 605 | 0.74 | 0.77 | 0.79 | 0.81 | 1062 |
|  | Eye (Lens) | 131 | 127 | 124 | 119 | 0.2 | 0.2 | 0.2 | 0.2 | 1076 |
|  | Eye (Sclera) | 1150 | 817 | 683 | 495 | 0.68 | 0.71 | 0.72 | 0.75 | 1032 |
|  | Eye *  (Vitreous Humor) | 76 | 74 | 73 | 72 | 1.5 | 1.5 | 1.5 | 1.5 | 1005 |
| 16 | Fat | 46.4 | 44 | 43 | 40 | 0.04 | 0.04 | 0.04 | 0.04 | 911 |
|  | SAT  (Subcutaneous Fat) |  |  |  |  |  |  |  |  |  |
| 17 | Hypophysis | 768 | 557 | 470 | 349 | 0.64 | 0.66 | 0.67 | 0.68 | 1053 |
|  | Pineal Body |  |  |  |  |  |  |  |  |  |
| 18 | Bone Marrow (Red) | 90.5 | 86 | 83 | 77 | 0.10 | 0.10 | 0.10 | 0.11 | 1029 |
